# How Playfulness Motivates – Putative Looping Effects of Autonomy and Surprise Revealed by Micro-Phenomenological Investigations

**DOI:** 10.3389/fpsyg.2018.01704

**Published:** 2018-09-11

**Authors:** Katrin S. Heimann, Andreas Roepstorff

**Affiliations:** Interacting Minds Centre, Aarhus University, Aarhus, Denmark

**Keywords:** playfulness, micro-phenomenology, motivation, gamification, autonomy, competence, creativity

## Abstract

Play and playfulness have repeatedly been suggested to promote learning and performance, also in environments traditionally not connotated with play. However, finding empirical evidence for these claims has been aggravated by the lack of a definition of play and playfulness fitting to this description. This paper proposes to consider playfulness as an attitude, mode or mental stance, that can be modulated independent of the activity pursued and of the general character of the person. It furthermore introduces the micro-phenomenological method to assess the process and outcome of such modulation. To explore this, we devised a simple building task in a controlled within-subject design, interviewing each participant on how they accomplished the task when asked to perform it so that it either felt playful or not playful. The outcomes of this data driven approach supported this notion of playfulness as a stance, and allowed for specific hypotheses about the temporal course and mechanisms of becoming playful. They suggest that an experience of autonomy and self-expression may be key to the success of the modulation. They furthermore indicate that the resulting playful state may allow for an exploratory engagement with materials that can lead to surprising results. Such unexpected results seem to enhance participants’ feeling of competence which, in turn, may increase the motivation for the task. We discuss these results within the framework of Deci and Ryan’s motivational theory and in relation to current research on gamification and learning.

## Introduction

*“P: I think … the most memorable thing was that I started smiling when you said it [*to be playful*]. And I felt like “Oh-yes!" And I felt like I could think about it and take my time instead of just rushing into it … So, I was... I was excited... but still… mh… calm and... or not calm... but, but like... more settled in a way… Before* [when advised not to be playful*]… I was very driven by… pressure, but maybe a little bit stressed and now I was just... driven by how I..., how I wanted to have fun and build these things and... and just play with it”* (Participant 3, talking about her experience to accomplish a building task in a playful stance).

Throughout decades, playing and being playful have been described as favorable or even conditional for humans’ well-being, performance, development, and even cultural evolution (see for example Murray, 1938; Huizinga, 1955; Bateson and Martin, 2013; Gray, 2013). Extending on these claims, the leading hypothesis of the quickly growing field of gamification is the assumption that play elicits high levels of motivation and creative behavior and that this can be utilized in education and work contexts to boost learning and performance. Studies exploring related hypotheses have risen to millions per year (see Scholar PLOTr^1^; key words play/playfulness/gamification and motivation/creativity, etc.), though with very mixed results (see for example Hamari et al., 2014; Hanus and Fox, 2015; Nicholson, 2015; Sailer et al., 2017). It has repeatedly been suggested that this ambiguity might not be due to low correlations, plainly. Rather the problem is likely to lie in a lack of agreement on how to actually define or identify the phenomena. Already Sutton Smith, reviewing play theories of the 100 years before 1997, summarized the state of the art as follows: *“We all play occasionally, and we all know what playing feels like. But when it comes to making theoretical statements about what play is, we fall into silliness. There is little agreement among us, and much ambiguity”* (Sutton-Smith, 2009, p. 1). Interestingly, this holds still true in present time, with fun researcher De Kowen stating: *“I’m beginning to think that I’ll never be able to define playfulness comprehensively enough to embrace it in its fullness. It’s just too diverse, too idiosyncratic, personal, profound to allow itself to be confined into anything satisfyingly definition-like. I’ve come to the conclusion that the best we can do is describe experiences, instances, moments in our lives that appear, in retrospect, at least, to have proven themselves unquestionably, undeniably, overwhelmingly playful”* (De Kowen, 2017).

This paper embraces De Kowen’s emphasis on the experience of being playful, but it does not concur with his claim that this experience does not allow for generalization and can only be captured anecdotally. Instead it presents an *empirical study* of the *experiential nature* of becoming playful. Our findings resonate with and extend on gamification research and allow to formulate specific hypotheses regarding the function of play and playfulness, in particular the connection between playfulness, motivation, and creativity.

### The Conceptual Challenge

There have been at least three different approaches guiding the many attempts to capture play and playfulness: by focusing on features of play/playful activities, by focusing on playfulness as a character trait and by focusing on playfulness as a frame of mind.

Firstly, most of the earlier definitions have attempted to identify specific features of activities that legitimize them to be called play or playful (for example play needs to be a spontaneous activity, not rulebound, non-literal, based on active engagement etc., see Blurton Jones, 1972; Reynolds, 1976; Rubin et al., 1983). However, empirical efforts to support the respective criteria have repeatedly failed (cf. Sutton-Smith and Kelly-Byrne, 1984).

**FIGURE 1:**
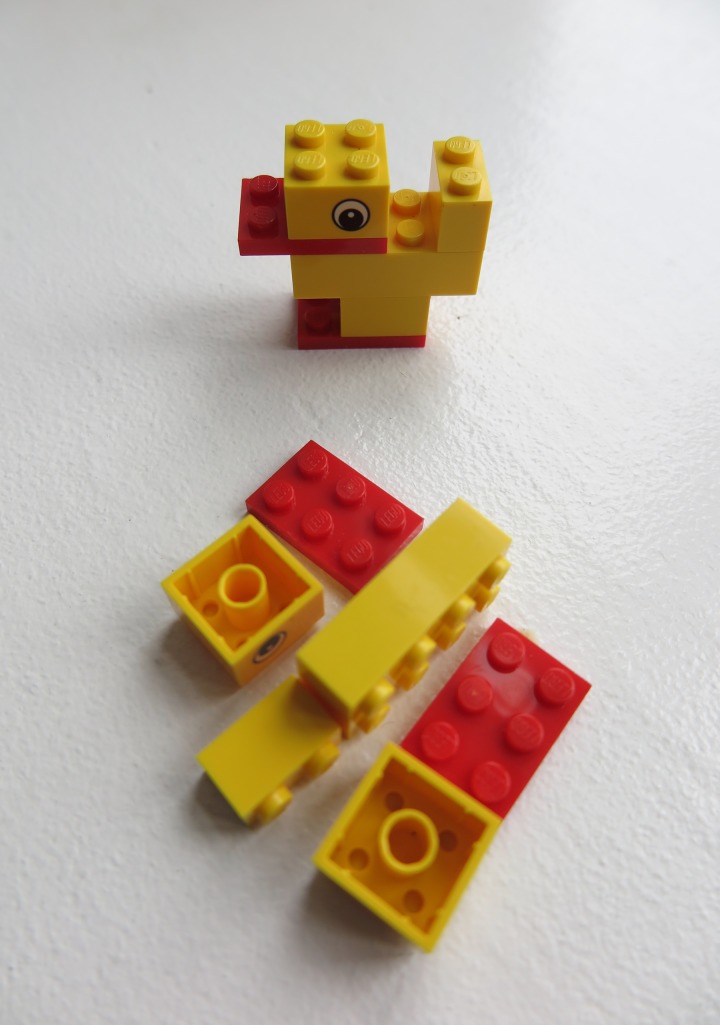
Prototype duck and LEGO bricks set used for the task.

Secondly, Lieberman (1965, 1966) was one of the first to shift attention from the *activity* to the *participant*, thus attempting to define playfulness as a character trait, with sub-traits such as cognitive spontaneity, physical spontaneity, social spontaneity, manifest joy and sense of humor (for recent work on this see Glynn and Webster, 1992; Proyer, 2012; Shen et al., 2014; see also Barnett, 2017 for a culture comparative approach).

However, much research in the fields of psychology, education, and in particular management and business administration seems to assume that one can have a more or less playful way to fulfill a task, independent of the activity and despite the fact that persons can be playful to a different degree. Building on the theories of Murray (1938); Caillois (1961) and Apter (1991), it has thus thirdly been proposed that playfulness should be conceptualized *“as the attitude of a person when he or she is engaged mentally and physically in the state of play. […] Any object can become a tool for play and any situation can be approached in a playful manner when the person is in such frame of mind”* (Arrasvuori et al., 2010, p. 2; see also Boberg et al., 2015); and elaborating on this: *“Playfulness is the expression of a universal capacity that can either be nurtured and encouraged or constrained and limited by both internal and environmental variables”* (Sanderson, 2010).

In fact, already the psychologist Susanna Millar has pleaded for such analytical shift, claiming that “*perhaps play is best used as an adverb; not as a name of a class or activities, nor as distinguished by the accompanying mood, but to describe how and under what conditions an action is performed […]”* (Millar, 1968).

### The Methodological Challenge

However, as argued by Sutton Smith, there is very little empirical work to further define this specific experiential state – and its relation to motivation and creativity:

*“What do the players reckon to be the character of and the reasons for their own participation? Obviously, there is not much research to be referred to here, although there is a considerable amount of anecdotal opinion to be cited”* (Sutton-Smith, 2009, p. 16).

The “obviously” in this quote most likely refers to the circumstance that introspection, the only way to assess subjective experiences, is not a method traditionally relied on within psychology. Two main reasons seem to be responsible for this circumstance. Firstly, subjects are often untrained to attend to and to linguistically express the micro-gestures in their minds. More importantly, they are even considered untrustworthy and highly susceptible sources of confabulation about their own mental actions (see for example Nisbett and Wilson, 1977; Johansson et al., 2005; but also Petitmengin et al., 2013 and, more generally, Jack and Roepstorff, 2003). Secondly, it poses a challenge for experimental psychology to derive generalizable information (numbers) from highly individual reports of experiences from complex, real life situations as the most commonly used quantitative methods, such as averaging, do not easily lend itself to this purpose. In consequence, the use of introspection has long been excluded in cognitive psychology as a means to study the human mind. However, as often pointed out, this is a curious circumstance given the fact that the harshest critics themselves not only commonly do trust their own judgments about their minds but also use these judgments – or “anecdotes” – to derive ideas and hypotheses for their experiments. As Jack and Roepstorff stress, the purpose cannot be to condemn introspection, but to validate and expand its use as a scientific tool (Jack and Roepstorff, 2003).

Micro-phenomenology (MP) is an interview and analysis approach explicitly developed for this purpose. It aims to facilitate the access to subjective experience and to analyze and represent it in a manner fitting to the scientific aim of generalization of results (for descriptions and validity tests of the method see Petitmengin-Peugeot, 1999; Petitmengin, 2006; Petitmengin and Bitbol, 2009; Bitbol and Petitmengin, 2013; Petitmengin et al., 2013, in preparation). It is also distinctly different from other assessments of experiences in so far as it looks at the detailed unfolding of an experience over time, rather than asking for overall characteristics (as assessed by questionnaires, etc., see for example Arrasvuori et al., 2010). This allows to explore (micro)-mechanisms and causalities otherwise unattended to, and it makes the approach a potentially valuable tool when trying to understand how a certain process might be facilitated or hindered – an obvious goal when thinking of the applicability of findings about playfulness for psychology, education or business. So far, micro-phenomenology has been applied to a wide range of topics, and the granted insights may have direct applicability in clinic, education, or organization. For example, it has been used to train epilepsy patients to detect (and treat) early signs of an arriving seizure, to help academics to understand and find first steps out of a writer’s block (Bojner Horwitz et al., 2013) and to assess and tackle some of the critical factors of overworking in executives (Créno and Cahour, 2015). It is furthermore used to reach a more fine-grained understanding of phenomena traditionally considered to be out of reach for empirical assessment such as emotions (as experiences, see for example Depraz et al., 2017).

### Aims of This Paper

In the following we will further present this method and our precise research design developed to explore the following questions:

- Is there empirical support for the hypothesis that playfulness can be considered an experiential stance that can be internally or externally manipulated?
- What are the experiential characteristics of the process of becoming playful in such way?
- Can experiential reports help to inform current discussions about the relation of play/playfulness and learning/performance?

## Materials and Methods

### Participants

Participants of this study were 22 young adults, 8 male, 14 female, 23.4 mean age (*SD* = 4.16), all but one with completed high school degree, 5 with further university degree. All were students of Vestjylland’s folk high school (Vestjyllands Hojskole) located in Velling, Denmark, a boarding school offering adult education for national and international students in several creative topics such as writing, dancing, theater, music as well as sustainable thinking and entrepreneurship^2^. The students were recruited on campus as volunteers after a 30-min interactive lecture introducing the method of micro-phenomenology as a tool of cognitive science, including a live interview about a simple spelling task with one of the teachers as interviewee. This setup allowed us to get participants motivated for the task and comfortable with the setup and the reflective requirements of the method.

All data were recorded within 1 week in May 2017, in a classroom of the Hojskole. Interviews were conducted in English by the same interviewer (Katrin Heimann). All students understood and spoke English on a level allowing them to study for a university degree. All participants received 150 DKK as reimbursement. They all gave written informed consent to procedure and data use and were debriefed after the experiment. The study was carried out in accordance with the recommendations of the guidelines of the Human Subjects Committee of the Cognition and Behaviour lab at Aarhus University and The Central Denmark Region Committees on Health Research Ethics. The protocol was approved by the Human Subjects Committee and exempted from need of approval by The Central Denmark Region Committees on Health Research Ethics.

### Data Recording Procedure

A micro-phenomenological interview begins with the elicitation of a particular singular experience. This is considered necessary to avoid a mere reproduction of information or knowledge about the phenomenon in focus (as triggered by simply asking what it means to get and be playful), and instead to foster access to an actual experience. For this purpose, MP always involves a clear reference event that the interviewee repeatedly is reminded to refer to. As the aim of our study was to explore the experiential process of becoming playful, we would ideally have referred to an instance when the participant had such an experience. However, asking for a personal memory fulfilling this condition would most likely have triggered experiences of very different nature across participants, which, in turn would have compromised the analysis of the data. We also wanted to avoid creating a “playful” experience by a prechosen context modulation (such as a “gamified” design) as for this we would have had to use (and therefore prime with) our anecdotal intuitions of how to get and be playful. We therefore decided for a controlled within-subject design that gave the participants the task to decide what it takes to become and be playful or not playful:

Briefly, for each participant we prepared six equal sets of six LEGO bricks, each set allowing to build a small duck.

From one set we built a prototype duck and placed it on the table. The other five sets were arranged in separate heaps in front of the participant. Each participant was then given one out of two tasks (order counterbalanced across participants):

Task (a): “I would now like you to build five LEGO ducks out of these sets. You can rebuild the prototype you see on the table or just build any duck or duck-like creature you like – that is up to you. The only thing that is really important for us and this experiment is that you do this as playfully as you can. Please find a way of doing it, so that it feels playful and nothing but playful.”

Task (b): “I would now like you to build five LEGO ducks out of these sets. You can rebuild the prototype you see on the table or just build any duck or duck-like creature you like – that is up to you. The only thing that is really important for us and this experiment is that you do it in a non-playful manner. Please find a way of doing it, so that it feels not playful at all.”

Notably, this design had only a minimal difference in instructions: the suggestion to build so that it feels as playful as possible or not playful at all. This contrast allowed us to explore whether adopting a stance of playfulness would allow for similar experiential qualities across participants, independent of the particular setting, activity or character of the participant (all constant across conditions).

If participants asked for further explication for the non-playful condition, we answered that it should rather feel like work. This occurred for the majority of participants, and we will discuss this as a possible priming issue in the last section of the paper.

After each building session, we ran a micro-phenomenological interview with the participants involving the following procedure: we started out by asking the participant the following question:

“Now, I would like you to go back to the moment in which I asked you *I would now like you to build five LEGO ducks out of these sets. You can rebuild the prototype you see on the table or just build any duck or duck-like creature you like – that is up to you. The only thing that is really important for me and this experiment is that you feel as playful as you can when doing it/you don’t feel playful at all when doing it.* Please take your time to go back to that moment and then tell me what you experienced. How did you accomplish this task?”

We used the principles of the micro-phenomenological interview technique to guide the interviewee to and through her experience avoiding to prime for certain answers or foster confabulation a posteriori. The main tool for this is that the interviewer, after the initial open question, only unfolds the answers of the participants in their own words. Thus, he mainly suggests to dive deeper into certain experiential episodes by repeating the participants’ phrasing and asking for further explication of the actual experience in all its dimensions. Most importantly, he avoids to prime the participant, e.g., by reformulating the experience according to his own experiences or prior knowledge or by asking for dimensions not mentioned by the participants himself. For more information see Petitmengin (2006).

In each interview, when the context allowed it, we furthermore asked “Did you manage to become playful/non-playful?” and – depending on the answer – “How did you experience it as playful/non-playful?” (and asking deeper into this as explained above). This allowed to explore the participants’ success in shifting his/her own inner stance and to explore how the result of the effort felt like with reference to the specific task.

Participants also filled out a questionnaire that explored basic demographics and asked about the overall experience in terms of *playfulness*, *enjoyment*, *fun*, and *duration perception*. We furthermore recorded videos, photographs of the ducks built, measured heart rate and galvanic skin response. Finally, we administered a short version of the Torrence creativity task post-experiment. The current paper only analyzes and discusses the interview data (based on video recordings) and the demographics. Complete interview data can be provided by the authors upon request.

### Data Analysis

Micro-phenomenology is based on the “elicitation interview” technique developed by Vermersch (1994) to help practitioners reflect their own praxis in the field. Thus, the original technique was designed to reveal individual thought and action processes to enhance each participants’ specific professional activities. More recently, micro-phenomenology has been adapted and refined for use in cognitive science, thus focusing more on how to generalize across participants. Obviously, this is dependent on a well-designed protocol. In this study we used the following four-step procedure based on Vermersch (1994, regarding the pre-analysis), Petitmengin (2001, 2006, 2009, regarding time analysis and analysis proper), and especially Depraz et al. (2017, regarding the generative aspects of the analysis proper and the constructive analysis).

(1) **Pre-analysis**: This step comprises
  (a) *transcription* of the data, providing the raw verbal text, “the Verbatim”;
  (b) direct and ongoing *commentaries* along the transcription;
  (c) *decoupling*, that is differentiating in the Verbatim between utterances describing actual lived experience and “satellite information” such as commentaries, theoretical generalizations, context information etc.

The end result of this analysis step consists of transcripts of the participants’ utterances (with interviewer’s speech removed) that describes only her lived experience (after satellite information has been removed).

(2) **Time-analysis**: This step consists in reorganizing the Verbatim of the lived experience so that it follows the timeline of the actual experience rather than the timeline of the interview.
(3) **Analysis proper**: This step involves analyzing the decoupled Verbatim with regard to structural categories, identified *a priori* on the basis of knowledge about dimensions of experience in general and *a posteriori* based on the experiential dimensions revealed in the actual data. The end result of this analysis step is a representation of the experience of each participant that explicates the type of experience each temporal phase contains: such as a memory, an imagination, a linguistic thought, a feeling etc. The following categories and subcategories guided our analysis in this phase (they were generally derived from Depraz et al., 2017, but see further specifications below):
  (a) *body* – referring to participants’ mentioning of (1) kinesthesis, that is bodily alterations/movements and, in our case, also distinct actions recalled; (2) perceptions, in our case including also more abstract perceptions, such as a pressure from outside etc., to the degree these are given a bodily basis.
  (b) *cognition* – comprising references to (1) memories, (2) imaginations, (3) attentional moves (focused/open), or (4) (meta) thought processes.
  (c) *feeling/emotion* – comprising references to affective reactions to the protocol that could be categorized as emotions such as happiness or as more diffuse states such as feeling ill at ease/stressed/bored etc. In the results we have grouped category (c) with category (a) as there was a direct link of perception and feeling.
(4) **Constructive analysis**: This step involves building a reduced model of the experience in general, keeping in mind the initial research questions. Thus, for each participant, we reviewed the results from the **Time Analysis** (2) as well as the **Analysis Proper** (3) both with respect to the two conditions and with respect to participants’ reports of whether they succeeded in entering the specific states. This allowed to highlight structural differences between the conditions (playful/non-playful). We also used this approach to explore if there was an order effect (when participants were first asked to build in a playful or in a non-playful way), but we did not find any systematic differences.

It is important to mention that the interviews and the analysis attempted to map the experiential process of becoming playful/non-playful, without explicitly frontloading specific theoretically motivated elements into the analysis. To the extent possible, the outcome of the analysis can therefore be considered data driven rather than hypothesis driven.

The primary aim was to analyze interviews to get insight into the experiential structure of becoming playful. However, we also used the videos to document the kind of products (duck-figures) produced in each condition and we evaluated the interviews in order to see if participants succeeded in manipulating the playfulness of their stance. In the following, we provide numbers (indicating how many participants out of 22 responded in a certain way) when generalizations across participants were possible, and we use direct quotes to either illustrate the generalizable phenomenology or to hypothesize about phenomena not touched upon in enough interviews to make an overall claim. The quotes are marked with PF (playful) and NPF (non-playful).

## Results

### Success of Modulation of Playfulness

The task to build while modulating one’s own feeling of playfulness seemed feasible for most participants. Only 3 out of 22 participants reported a difficulty to achieve a playful stance. To build in a non-playful stance seemed to be slightly harder with 12 participants reporting difficulties (though not failures). As the experiential analysis revealed, this might be related to the use of the LEGO material facilitating a playful stance rather than a non-playful stance. We will refer to these instances with more detail below.

### Experiential Structure and Products

In total, we identified four different phases of the experience in general:

(1) Modulation of the playful stance
(2) Imaginative building preparations
(3) Building
(4) Product evaluation.

While there might be other phases involved in other tasks, we assume that the ones detected here cover more general process characteristics of any such task, such as a preparation phases (1 and 2), a conduct phase (3) and an evaluation phase (4). In the following we will describe each of the phases with respect to the experiential categories as defined above. To give a full picture, we also include third-person reports of building products or behavior in these otherwise first-person descriptions (differences clearly marked in the text).

#### Modulation of Playful Stance

The majority of participants reported about thoughts about how to modulate their own playfulness that occurred to them right after they received the task. Similar micro-experiences also happened in later phases of the task, seemingly as the result of participants evaluating the state achieved so far and possibly trying to correct it to better fit the goal (that is to be playful/non-playful). In the following, we report about the precise content and phenomenology of this phase using the categories described above.

##### Cognition, meta-thoughts/inner speech

Almost all participants reported the occurrence of conscious linguistic thoughts about the meaning of the task given. Strikingly, the vast majority of these reflections addressed a modulation of the feeling of autonomy as well as certain directions for how and what to build. In the playful condition, participants mentioned that the demand to be playful essentially meant them to be set free to do whatever they wanted to (14 participants) or to create something clearly inspired by their own ideas or intuitions rather than any pre-given options (7 participants). In six cases, these thoughts were reported to have come to participants as direct inner speech, giving the claims a very distinct and confident character, see for example:

“P: Ehm… I remember smiling eh… first when you said it... that I was about to play... I immediately started smiling... and eh... then I thought “Okay now I can do whatever I want and I can take my time” (P3, PF).

Fifteen participants in total, still in the playful condition, furthermore described that they experienced an urge to be creative or to build different ducks – at least as long that this would not lead to a stressful experience:

“P: I think my first thought was to like make five different, because I found that kind of playful before … but then I… also…thought back to that experience and it was also… actually kind of… work-like I think, because it was also bit stressful, you know, to have to make five different... that look like a duck... so, mh… I think I just…to make it really playful it had to be… like I could do it different, but it didn’t have to be like five different ducks…” (P7, PF).

In contrast, in the non-playful condition, 16 participants reported thoughts indicating that experiencing constraints and stress was taken as essential for fulfilling the condition. This included a constraint of building as fast as possible (time pressure) and of fulfilling certain expectations on how the product should look like (evaluation pressure). See for example:

“P: I thought a lot more about speed... And I was… much more worried with what the two of you would think… Ehm… yeah, but speed was the first thing that popped into my mind, in order to making it feel more like work, and then at some point correctness” (P6, NPF, focus on time pressure).

or also:

“P: I was thinking about that I should do this with more than five ducks and just keep on going. And that it needed to be the same as this one [points at prototype duck] … it’s like, I couldn’t use my imagination and I just needed to produce... in the right way” (P4, NPF, focus on evaluation pressure).

The last quote is also an example of one of 13 participants who explicitly mentioned having thought that the task to be non-playful directly implied copying the prototype duck. Elaborations from four of these participants suggested that this might be related to the expectation that copying is a meaningless and boring activity.

Notably, still in the non-playful condition, two participants reported these thoughts occurring to them in the form of inner speech. However, the experience seemed very different from that of the playful mode, in which participants reported hearing their own voice contently noting the freedom given by the instructions (see above). In contrast, in the non-playful condition participants reported a constant reminder from “a” (thus not necessarily their own) voice asking them to get going. See for example:

“P: It’s just like... with like working I... I kind of heard the sound like in the military… like “Working! Go! [tuftuf] […] I just… remember there was this like voice “come on... make ducks” like a voice saying like “come on” or something like…

I: Okay. There was a voice that told you to get... get on with it?

P: Yeah, not…not like pep-talkish, but like “do it…do it”” (P19, NPF).

##### Body, perceptions; feeling/emotion

Strikingly, many participants reported that their thoughts and inner voices were accompanied by immediate bodily reactions fitting the assumed affordances of the conditions. Of the participants indicating that they reached a playful stance, nine mentioned the immediate *feeling* of being relieved of any or certain obligations, as well as being encouraged to explore and enjoy themselves. See for example:

“P: Ehm... well, I think the… the most memorable thing was that I started smiling when you said it. And I felt like oh – yes! And like, I didn’t… and also yeah, I like I… I felt like I could think about it and take my time instead of just rushing into it. So, I was… I was excited... but... but still mh… calm and... or not calm... but, but like… more… more settled in a way…”(P3, PF).

In contrast, of the participants indicating that they reached a non-playful stance, 16 reported the *feeling* of an obligation to fulfill the task in one or the other way, causing them stress and partly also boredom:

“P: It was maybe a bit more stressful, because I felt I should do it in the right way… So I needed to [gestures repetition]… get over, do it again, if it… if it wasn’t right” (P4, NPF).

This happened even when participants explicitly noted that they themselves were the ones actually being in charge about the actions to be performed. See for example:

“P: You said that it was my decision. You said that… [I: mh] I could [I: yeah] make them however I [I: yeah] wanted, but… when you said that it was work and not play, I automatically assumed that I was creating a product that was… supposed to look like the original model” (P3, NPF).

Notably, the three participants that admitted difficulties in reaching a playful stance connected this to the feeling of heteronomy (that is being determined by other circumstances than own will). While one participant explained that she also tried to accomplish the task “correctly,” a demand which to her compromised her feeling of playfulness, the other two explicitly stated that being given a task or being in an experimental situation in general had hindered them reaching a real playful stance.

##### Cognition, memories

12 participants reported the occurrence of memories directly related to the modulation of playfulness.

In the playful condition, these were without exceptions memories of play during own childhood or of playing (as an adult) with children; while in the non-playful condition, participants recalled mostly memories of prior or current work places. Underlining this difference again, the five participants, for whom the building activity in the non-playful condition evoked memories of play in childhood, reported some degree of difficulty achieving the non-playful state. See for example:

“I: Did feel like working actually? P: Mmh… kind of yeah… but… I still have a lot of memories with LEGO... So, in that way it was still... I mean I remember ehm… these brochures with different things you could build… like big castles and stuff… And I remember my father and I playing with them and... and building them according to these brochures. So, in that way it... it had a hint of that and it... it like... yeah but...but mostly it felt like a factory job to a degree” (P3, NPF).

Conditions further differed regarding the occurrence of those memories. In the task to build playfully, participants reported memories happening like a flashback, without conscious intent. In contrast, for at least four cases in the non-playful condition, the memory seemed to have been intently evoked to help the elicitation of a certain inner stance. The following quote shows how such effort facilitated getting into a work mood in the task given:

“P: Okay... I… was thinking about eh… my university… because, I am studying architecture and we usually have to build something... models, yeah… and I tried to be in that position… […]

I imagined, if I were… eh... in the work place… with my… with my... partners and… [laughs] I don’t know… I... I tried to imagine it…the surroundings. And… (pauses)

I: How was it? How… How was that situation for you?

P: It was so many people in my room and… all of them… did their own work and… and their own eh… projects

I: Mh… And how did it feel to be there?

P::...Like I needed to do it

I: You needed to do what?

P: To... to build ducks, but not exactly the same ducks.

I: Mh. So, but how did you... So, this was a transfer from the university context to here?

P: Yes” (P 3, NPF).

##### Cognition, attentional move

In both conditions, participants reported a heightened attention for the task. However, while in the playful condition such state seemed to come naturally with the building, in the non-playful condition, 4 participants reported this move as an effortful activity, demanded by the task:

“I: Do you remember anything else of your body feeling? Or of your thoughts when you were building the ducks? P: Ehm… I... I…I think it... this... this whole being drawn in… I am building something, which sucks me in […] it takes my focus” (P2, PF, no effort).

“Well I had to do it like working...so I tried to be more focused… like saying, at least when doing the first copy… like… “okay.” I... I would normally just put it together, but here I am like... “okay… take it step by step... placing the first brick… then the next one... “and building up from there… bit more metho... dolo... gically…” (P1, NPF, effort).

To sum up, participants’ descriptions indicated that from the very start of the experience, the different tasks triggered micro-experiences clearly differing between conditions regarding content and experience. In the playful condition participants indicated pleasant conscious thoughts about the association of such mood with the experience of autonomy and creative production. Noteworthy, such thoughts seemed to be accompanied by immediate feelings fitting these requirements, such as relief, freedom, inspiration, and enjoyment. Such feelings might have been facilitated by spontaneous memories to pleasant childhood play experiences and an effortless raise of attention for the task. In the non-playful condition on the other hand, participants indicated demanding conscious thoughts about the association of such mood with the experience of outer and inner constraints, pressure and meaningless repetitive actions. Such thoughts seemed to be accompanied by immediate feelings fitting these requirements, such as heteronomy, stress, and boredom. These feelings might have been facilitated by intently evoked memories of former working places fulfilling these conditions and the perceived strain to constantly focus attention on the task.

#### Imaginative Building Preparations

This phase was derived from a number of participants who referred in their experiential reports to mental imaginations that preceded the duck building. Further instances were found within the building phase in which participants used such as an inspirational as well as corrective tool for their activity.

##### Cognition, imaginations

Five participants reported visual imaginations of ducks as a perceived mean to facilitate, inform, or inspire the building task faced. See for example:

“P: I visualized, I think, eh… duck... ducklings actually. Eh... a few weeks ago Justine had some ducklings and that’s were… those were the first that came into the mind – my mind. And then some images of the rattle Donald Duck. And ehh… yeah… then I just tried to put together the bricks…” (P10, PF).

While some of such visualizations were of very concrete character, others seem to be more schematic. Thus, participant 10 reported shortly imagining the ducklings of a neighbor that he had seen the week before, and participants 5 and 10 both recalled in a moment thinking about the comic figure Donald Duck, hissing. On the other hand, participants 1 and 8 indicated that they were rather imagining different “duck positions,” without being able to precisely describe the visualizations connected, but rather calling them “schematic concepts.”

In general, reports of imaginative experiences seemed more common in the playful condition. The only case (out of five in total) that was reported for the non-playful condition was one of “schematic character” – possibly however, this might be at least connected to if not caused by the bias for copying the prototype in the non-playful condition, which naturally affords less creative planning.

##### Feeling/emotions

Interestingly, three out of the five participants reporting visual imaginations expressed some kind of emotional dissatisfaction quickly developing along with this experience. The reason for this seemed to lie in the circumstance that the translation of the mental images into a LEGO brick construction posed a strong, often unsolvable challenge. That is, participants mentioned that they were not able to live up to the pictures they drew in their head when dealing with the actual bricks.

The report of one of the participants furthermore indicated that this feeling might be more prominent in the non-playful condition, while in the playful condition participants might be able to somehow “let go” of the self-opposed matching task, allowing anything to happen:

“P: I tried to use these bricks [points with both index finger at third duck] in order to build something that would look like a duck flying. And… well, I encountered the same problem again (like in the non- playful condition, added by authors). I couldn’t get the image to fit… and sort of doing the working thing, I’ve really tried to, like... “okay this image needs… to fit…” and like, make a model… Then here it was more like experimental… Just going, could I do this, that or something else” (P1, PF).

To sum up, participants’ descriptions indicated that the different tasks triggered imaginative efforts clearly differing between conditions regarding content and experience. In the playful condition several participants indicated concrete associations to ducks seen in different contexts (of real life or illustrations seen). The translation of such imaginations into the building material was experienced as not very feasible, however this failure did not lead to frustration but rather allowed to open for the interaction with the material. In contrast, in the non-playful condition only one participant indicated an imagination preceding the building phase. However, this association to be of a schematic character, and the difficulty to translate such image into the building material was experienced as frustrating, leading to repetitive trials, rather than opening for creative production.

#### Building

With this phase we refer to any micro-experiences that accompanied the actual building of ducks or “duck-like” creatures.

We begin by a description of the building outcomes, before elaborating on the experiential reports of the participants:

In the playful condition, only three participants built the same duck again and again, while the other 19 participants built five ducks that did not look alike (though in four participants one of these was an exemplar of the prototype).

In contrast, in the non-playful condition, 10 out of the 22 participants built prototype ducks only, one participant always built the same duck different from the prototype, nine built one to three prototypes, while the rest of the ducks differed to a smaller or bigger degree, and only two participants built five different ducks. Three of the participants, who in the non-playful condition did not restrict themselves to copying, explicitly stated that this was due to a strategy change happening while building. Interestingly, they indicated that the experience was getting too easy to be still considered work or a job:

“P: I think that when I had built that one and it was so easy, then I was just like… mh… it’s… doesn’t really feel like I have… done like… a job, if I just do five of those… Cause it’s... [shrugs with shoulders]” (P7).

It is possible that these cases are caused by the identification of a non-playful stance with a work attitude. We will get back to this in the discussion.

It is also worth noting that in the non-playful condition, all participants built *ducks* – as instructed. By contrast, in the playful condition, four of the participants explicitly reported having constructed *something else* than ducks:

“P: I just started trying to… look what came out of it. I didn’t really think about it… but still… ehm… it’s a bit difficult to explain, I feel, but mh... I... because I did it mostly like: I didn’t think so much about it, but at the same time I was trying to be maybe a little, or maybe a bit creative.

I: How did you do that to be creative with that?

P: Mh. to… build something that looks like an animal. I was going for... making animals.

I: You were going for making animals. Not ducks actually. Just animals?

P: Yeah […]

I: Did you think about a specific animal here or how did you…? [points at duck3]

P: A little bit. I don’t remember the... it’s not an “animal-animal,” but there is... so, there is this game… […]where they eat ehm... [gestures eating] It’s “pacman” I think. Yeah. I thought a bit about that

I: Mh. Okay. Did you... was that eh... eh... a thought that you had before building it, or? During or?

P: Eh... after

I: Afterward?

P: Yeah

I: You saw it and then you thought about pacman?

P: [nods]” (P21, PF).

The quote indicates that participants might not initially have intended to break the rule (of building ducks). It rather indicates that participants, when being playful, did not have a precise idea of what (else than ducks) to build in the first place and that the new products were a result of the playful stance which made them stay open to the process:

“P: Mh... yeah, it feels playful that there isn’t like any rules. That can I, like, create whatever… I want to… and that... was also playful that you didn’t, like [picks up duck1], necessarily, had to, like, see what it is [laughs]. What animal… So that I could like… ehm… change my mind about what it is... this was a man [picks up duck4] or… some other animal...” (P23, PF).

Such explorative mood seemed to be enhanced by the LEGO material that fostered a trial and error approach:

“*P: Well... It’s LEGO… so it makes it bit hard to like really feel working with it… […] it’s... hm – it’s maybe not a clear thought I had, but it pretty quickly once I got my hands on, it… was... well… somewhat natural just to fiddle with it…I played a lot with LEGO as a child…so it’s sort of like… fiddle hand… and… yeah...[…] also maybe it’s because it’s such an easy task… I just like want to do it…rather then put too much thinking into it” (P1, NPF).*

The same participant also indicated that the LEGO material provided enforced this approach by making it difficult to fulfill a precisely set goal, such as an interior image of a duck. This might have helped to create an open space for the unexpected to happen:

“I think… I wanted to do a flying duck… but that one [clearing throat and points at duck 3] ended up being, like, a duck standing with its wings out. That one [points at duck 4] ended up being like a duck in the water…. with wings out. And that [points at duck 5] might be a flying duck – or something else… I am not sure. [P softly laughing]

I: And you thought about these things, while you were building this?

P: Yeah, so… oh… no, not while building – at some point there was just like… I needed one brick more or something and I couldn’t really find it and then was just like… okay… And... then the model changed… Like, I sort of thought, ok, maybe it can become what I want it to be, but now it looks like this. – And…I am not sure if it was like… once the last brick was sitting or when I was done… but it sort of was like, okay that’s what it is” (P 1, PF).

The following experiential descriptions extend on this finding.

##### Cognition, metathoughts/inner speech

We consistently found conscious linguistic thoughts guiding the building experience. In particular in the playful condition, participants seemed to use this tool to manage a delicate balance of creative pleasure and stress:

“P: I think I... ehm... I just didn’t want it to… to make it a… a… more difficult task. I think it would re...quire... too much ehh… [I: mh] creativity from my site and I thought this was like the safe way and the... it wouldn’t feel like... I think I easily would feel like... that I did it wrong, if I did anything else. […]

“I kept telling myself that... “But it’s not a competition!” to make myself feel more... calm about it.

[…] Maybe… playful and doing things a 100% correct... is not super... ehh...” (P6, PF).

##### Body, perception; Feelings/emotion

Further qualifying the experience of the building phase in the playful condition, 14 out of 22 participants reported feeling relaxed, free and having fun. One participant even replaced the word playful by “joyful” in the conversation:

“P: So… now when it has to be more joyful, so I don’t have to think about it, but I already get the basics. Or I tried it in fact… So now it’s… it’s like I know this thing, but I can do whatever I want

[…] I: So, you said you could just do whatever you want. And when… you always say eh… “joyful” so is that an equivalent for you to playful?

P: Yeah, yeah!” (P11, PF).

On the other hand, 10 out of 22 participants reported negative feelings arising from the non-playful building, with boredom and stress being most prominent.

“P: This was just boring and... I... felt this pressure, you know, …I have to do this, because this is a working task and I find no ehh... happiness in this one… (I felt) a challenge also, because it’s work so I should know how to build this... probably build the same for 8 h... 1000s of times every day. And eh... I lost most of the interest in [reaches for duck 3 and picks it up] how to... build this, because I… I knew it” (P2, NPF).

Furthermore, in the playful condition, two participants pointed toward an aesthetic quality of LEGO: its particularly pleasant tactual experience.

“I like in LEGO that they… [picks up prototype] the machine… the... this fabrication machine…it’s so perfect you know. They always... fit [presses head down... into another and they... I enjoy eh... this feeling when it... you know? … sticks… it’s so smooth and it has a little tension I... I like when they [claps hands together] go…together.”(P2, PF).

“And so it’s lots of positive associations with the touch and the feel of it and… the feeling when… [mimics pressing bricks on top of each other] the sound and the feeling of it connect.

It’s like... ‘cause there is a feeling, there is a sound. It’s like, if you… if you close a book [gestures closing a book]. It can be a... a very subtle sound… It can be like “ahh”… It’s a really good feeling” (P22, PF).

In contrast, this pleasure was not found in the non-playful condition:

“I lost most of the interest in [reaches for duck 3 and picks it up] how to… build this, because I... I knew it. I didn’t find it eeh... pleasant anymore to put together the bricks... I just pressed [demonstrates] on the top... I didn’t feel, you know... anymore this… pleasure… of clicking them together” (P2, NPF).

The interview with participant four suggests that it is the experienced evaluation pressure evoked by the non-playful condition that hindered such an experience:

“P: I was maybe a bit more stressful, because I felt I should do it in the right way. So, I needed to [gestures repetition]... get over if it...if it wasn’t right.

I: And how does it feel to be more stressful?

P: Mh… not nice [laughs] … yeah...it makes it more difficult to build, actually, when you more stressful, because you are not relaxing…so it was a bit di... more difficult to do it. Yeah.

I: How was that?

P: Mhm… Just really, if… you put the brick in the wrong place you had to move it and I think I could feel it in my fingers. They were less relaxed…So it was more difficult actually to put a brick in the right place.

I: Okay… in the way that you grasped them?

P: Yeah, or put it” (P4, NPF).

To sum up, participants’ products and descriptions indicated that, also within the building action, the different tasks triggered micro-experiences clearly differing between conditions regarding content and experience.

The majority of participants in the playful condition built five different constructions, some of which did not even represent ducks. Rather than the outcome of a conscious strategy, this appeared at least partly to be the result of a distinct openness to the process induced by the autonomous stance taken. Participants’ reports furthermore suggested that such openness might have been facilitated by a conscious care to keep up a good mood and a low stress level. This mood management appears in turn to have allowed for a higher sensibility toward the building material, by facilitating a perception of its aesthetic qualities, which again allowed for further exploration and openness toward the process.

In the non-playful condition on the other hand, the majority of participants produced several copies of one and the same construction. Furthermore 10 out of 22 participants reported negative feelings, such as stress and boredom, arising from such building. Interviews also indicated that such feelings might have reduced the sensibility for the material, by this further narrowing the action space available.

#### Product Evaluation

We identified this phase based on a number of participants reporting detailed inner reactions to their own finished products. While most participants built the ducks one at a time, the evaluation most often referred to was the one at the very end of the building face, looking at all their products together.

##### Body, kinesthesis/perception

After having finished the last duck, participants often took a moment to look at their products (we did see at least 12 doing so clearly in the video), partly even rearranging them (six participants), before telling the experimenter that they had finished the task. This behavior was particularly obvious in the playful condition (comprising all of the 12 clear cases), possibly influenced by participants building more diverse ducks (see above).

##### Feeling/emotion

Participants’ reports of these moments were marked by descriptions of feeling/emotion, with joy and surprise being the most prominent. In fact, four participants stressed that they had not known that they would be able to produce such products:

“I: And when you saw it ready… how did you feel?

P: I felt ehm… satisfied [laughs]… yeah... I was actually a bit surprised, that I… I did sort of …got something like that” (P8, PF).

In the non-playful condition, on the other hand, one participant pointed out that not being surprised was essential for this condition:

“P: I was thinking about that I should do this with more than five ducks and just keep on going [And that it needed to be the same as this one [points at prototype duck] … it’s like, I couldn’t use my imagination and I just needed to produce... in the right way.

[…] I think because than I think it was... would be boring… just like, for a long time p... period...

[…] There wouldn’t be any surprises along the way. It was just be the same again and again”(P4, NPF).

To sum up, interviews indicated that also participants’ experiences with their finished products clearly differed between conditions regarding content and experience. In the playful condition, the majority of participants spent obvious time on looking at their products and reported positive emotional reactions to such (satisfaction and surprise), while in the playful condition, such reports were much more scarce and rather marked by negative expressions (boredom etc.).

In the following we will review these findings regarding the whole experience and reflect them in the light of current discussions in the field of gamification.

## Discussion

Our study aimed to assess three questions: (a) Can we find empirical support for the proposition to look at play and playfulness as a stance or state of mind that can be modulated by internal or external variables? (b) What are the experiential characteristics of the process of becoming playful in such way? (c) Can such investigations inform current discussions about the relation of play/playfulness and learning/performance?

To assess this, we used a controlled within-subject design, interviewing each participant on how they accomplished a building task when asked to perform it so that it either felt playful or not playful. In the following we discuss these questions in turn.

### Playfulness as a State of Mind

The interviews indicated that participants in general could relate to the instructions and were able to enter a playful stance, with 19 out of 22 participants reporting that they managed to do so. It seemed slightly harder for them to achieve a non-playful state, with 12 participants reporting difficulties to do so. Close evaluation of the interviews suggested that difficulty to get in a non-playful state might be an effect of the LEGO material provided that carried too many associations to and memories of play. Some participants, albeit less pronounced, expressed difficulty in becoming playful; this appeared bound to the experimental situation, which restricted participants’ feeling of autonomy. Taken together these findings support the claim that participants could assume a particular playful stance, generated by a voluntary internal modulation. They further show that external variables (e.g., experimental context and setup) must support this internal effort for it to be realized completely. In particular, they must support a degree of autonomy which playfulness seems to require.

### Experiential Qualities

This conclusion is supported by the most striking finding in the micro-phenomenological assessment: a feeling of autonomy seems to be constitutional for the ability to modulate playfulness. 14 participants reported immediately thinking that this task essentially meant being free to build what they wanted to – an indication of the importance of freedom of choice. Seven furthermore reported that they felt encouraged to create something “with their own minds” – an indication that the personal meaning of the task was essential to them feeling playful.

This contrasts starkly with the description of 16 participants in the non-playful condition who reported thinking about or immediately feeling constraints regarding their building actions and/or a deprivation of meaning regarding the task. One participant even explicitly referred to the quasi-sensation of a voice telling her to “do this, do this” which vividly exemplifies the lack of autonomy experienced.

Interviews furthermore indicated a range of strategies that participants considered as key in achieving and maintaining one or the other of the two stances explored: in particular, several participants recalled the need to actively use attention and memories to get into a non-playful stance, while the transition to be playful seemed to happen almost effortless, facilitated by spontaneous flashbacks into childhood or joyful situations of playing with children. There were indications though that this difference might be linked to the material used in the building task as we will discuss later.

Our data further revealed that the two conditions differed in what participants considered to be appropriate building products: 13 participants, when advised to be playful, recalled explicit thoughts about building five different ducks in the playful condition. This seemed to happen with the intention to enhance self-expression and creativity and was pursued as long as this task did not imply too much stress – a feeling apparently connected with work. In contrast to this, when requested to act non-playfully, 15 participants reported an inner advice to only copy ducks. The purpose of this seemed to be to create an atmosphere of restriction, meaninglessness, and boredom – and it was pursued as long as this task did not get too easy – a circumstance seemingly excluding an activity to be considered as work.

Participants’ reports correlated strongly with participants’ final products in the two conditions: the majority of participants in the playful condition built five different constructions, while in the non-playful condition, the majority produced several copies of one and the same construction. Notably, four participants in the playful condition in fact reported not building “ducks” at all, that is, their drive for freedom and creativity made them even ignore a critical part of the (very minimal) task instruction.

The interviews suggest that this higher expression of creativity in the playful condition may be the outcome of a dynamic process set in motion by taking an autonomous stance: freed from specific constraints and goals, participants seem to enter a curiosity driven interaction with the material, which allows for an unknown outcome to occur. This process might have been enhanced by an aesthetic way of perceiving the building material, enforcing an exploratory approach due to the sensory and reflective pleasure involved. Interestingly, this process may result in unexpected products, and the realization of this appears to enhance participants’ feeling of competence.

These findings suggest that participants entered the playful condition by a contextual reinterpretation of the situation. This involved allowing oneself to feel autonomous and be exploratory without these self-imposed directives becoming constraints and stressful factors. In stark contrast, entering a non-playful condition was achieved by establishing a context of self-imposed constraints (e.g., time pressure or evaluation) and by reducing exploration, surprise and enjoyment to a minimum. This also appeared to reduce their experienced feelings of competence and motivational drive. Tellingly, if these constraints were not met, participants reported that their experience did not meet the requirements of the non-playful task.

The importance of autonomy as well as enjoyment for play and playfulness have been noted before, for instance in attempts to identify play with reference to specific features of the activity or the players (see section “Introduction”). However, these approaches do usually not not make any claims about the status and role of such features in the temporal course of modulating and being in a playful stance. To take one example, Bateson and Martin (2013) list that play behavior must be (a) spontaneous, intrinsically motivated and fun and (b) the players free from illness or stress (see also Fagen, 1981; Burghardt, 2005). They furthermore claim that “playful play is accompanied by a particular positive mood state in which the individual is more inclined to behave (and in the case of humans, think) in a spontaneous and flexible way.” The psychologist Erikson offers a temporally more detailed model, however from a developmental perspective. He argues for a “play stage” in human development that is entered right after the “early childhood stage,” which is aimed at achieving autonomy. Given the development of autonomy in the play stage, the child can learn to take initiative and engage in a world shared with others which is accompanied by a sense of mastery (see Erikson, 1959; as well as Proyer, 2018). We believe that our data offers a refined picture of how these mechanisms unfold in the distinct time course of each single playful experience. They show how a momentary modulation of autonomy influences consequent behavior and experience, how mood management and manipulation are involved in this process and how this affect the feeling of mastery/competence. These findings can be closely connected with current research about the relation between play and motivation and learning.

### Playfulness and Learning

Our findings bear striking similarity to those described in one of the most influential theories of motivation: the psychologically oriented Self-Determination-Theory by Deci and Ryan (2000, 2015). Briefly, Deci and Ryan identified a spectrum of motivation from the autonomous to the controlled, with the two extremes being *intrinsic motivation* (most autonomous), when people find an activity naturally interesting and enjoyable, and *extrinsic motivation*, when people are not interested in the activity itself, but in a consequence of their engagement such as a reward (most controlled). In Deci and Ryan’s analysis, establishing intrinsic motivation is preferred to extrinsic motivation in particular with respect to learning, as it is not dependent on a supporting framework. The framework furthermore claims that originally extrinsic motives may be internalized if they serve fulfillment of human’s basic and innate psychological needs (Deci and Ryan, 1985; Ryan and Deci, 2000). Specifically, intrinsic motivation is increased by satisfaction of our natural need for *autonomy* (comprising freedom of choice and integration of values); our need for *competence* (the propensity to have an effect on the environment as well as to attain valued outcomes within it); and our need for *relatedness* (a natural sense of belonging to the environment and the people that are with us). In simple words: we won’t enjoy – and therefore be naturally interested in – an activity, if we are coerced to do it, not convinced about its value, unable to master it, or if it deprives us of our feeling of belonging. On the other hand, the more autonomous, competent and related we feel when doing it, the stronger our intrinsic motivation can get.

Seen in this light, our findings suggest that creating the conditions to get playful means creating conditions to get intrinsically motivated. We found that participants intuitively used a contextual reinterpretation to modulate their degree of autonomy when asked to modulate their stance of playfulness. Further, they reported that the success of the modulation of playfulness depended on the success of the modulation of autonomy. In particular, when the outer context was perceived to oppose a feeling of autonomy, participants reported difficulties in becoming playful. Indeed, allowing for a playful stance crucially seems to depend on the creation of a sense of autonomy. This suggests that situations lacking autonomy will not be experienced as playful, and that designing the specifics of the context (the organizational environment etc.) may be crucial for allowing such autonomy.

Our findings further present a possible mechanism for how the experience of autonomy may evoke a feeling of competence: the diversity of ducks built in the playful condition was experienced as a result of the openness of the process, created by the autonomous position. Many participants reported a dynamic interaction with the building material, at times experienced as aesthetic, which allowed for an exploration of its possibilities. Many explicitly mentioned to be surprised by the results of their activity, stressing that they had not been aware of their own capacity to build so creatively. We suggest that this represents a concrete experience of competence, thus providing the second key component of intrinsic motivation.

### Playfulness Versus Gamification

Finally, our findings may throw new light on key problems faced within the field of gamification. Briefly, gamification is the overarching term for the approach to introduce “*game design elements within non-game contexts*” with the explicit aim to achieve levels of motivation “*as high as* *for playing video games*” for a task that is considered difficult to initiate (Deterding et al., 2011, p. 1). However, a number of studies have indicated that the approach may face some fundamental issues. Though the majority of research exploring the effect of gamification on motivation have found more positive than negative or null effects (Hamari et al., 2014; Seaborn and Fels, 2015), it has been claimed that the main kind of motivation established in gamification is extrinsic, rather than intrinsic, and that the effects may thus not transfer outside the specific context (Nicholson, 2015). It has even been warned that designs that are more extrinsically motivating might risk to replace intrinsic drives on the long run, thus creating a constant dependence on reward structures and other forms of extrinsic evaluation (Deci, 1971, 1972; Deci et al., 1999; Kohn, 1999). Thus, Hanus and Fox (2015) showed that gamification in a classroom led to lower performance compared to a group attending non-gamified tuition and that this result was mediated by the lower level of intrinsic motivation of the gamified tuition group. Most interestingly for us, it has been suggested that the main cause of this circumstance may be that gamification, by focusing on game design elements, focuses on creating a with play associated activity rather than a “playful stance” *per se* (Deterding, 2010; Nicholson, 2015). However, only the latter might be effective in enhancing intrinsic motivation. Indeed, Deterding (2016) showed that some aspects of games, such as playing in a team, might lead to the feeling of obligation, a loss of playfulness and therefore reduced motivation to play (Deterding, 2016).

Our results support and extend on this idea: they provide strong support for the hypothesis that allowing for a stance of playfulness may be an effective way to increase intrinsic motivation. Furthermore, they generate specific hypotheses about what processes one should pay attention to, if intending to design playful experiences.

Experiencing autonomy, that is, the feeling of freedom and meaningfulness of own actions, seems key to adopting a playful stance. We found that our participants had internal means to modulate this feeling – but there may be constraints to this, due to differences of context as well as personal capacity.

There appears to be a thin line between empowering self-determination and the experience of stress due to self-imposed expectations. It is possible, that different capacities to adapt own expectations accordingly affect the capacity of being playful and that training focusing on sustainable self-management might thus support playfulness.

The situational context seems critical for achieving the experience of autonomy. In particular, participants mentioned that the experimental setting in itself was a restriction. This may also apply in educational as well as professional environments. Our findings do not suggest a solution for this problem, though they indicate that some individuals could overcome this in time. There is scope to research this further.

Our findings highlight the importance of the properties of the physical material involved in the experience – in our case exemplified by the LEGO bricks provided. It seems that a material might differentially foster explorative behavior due to (a) its abstractness, that is its resistance to be guided by mental imaginations and (b) its sensual, aesthetic properties and design. Our findings suggest that the abstract, modular qualities of the material provided made a direct translation from a mental image into the model difficult and that this increased the explorative potential. This was further supported by sensual pleasures that may enhance further exploration. However, due to the small sample size, these hypotheses should be followed up by research.

Lastly our findings indicate that autonomy and competence (as two critical components of intrinsic motivation) may in some instances stand in a causal relationship. Thus, a feeling of competence can be evoked by unforeseen products of the explorative process facilitated by an autonomic, playful stance. This proposes a putative looping effect of autonomy and surprise, which may be critical in supporting intrinsic motivation during a playful stance. Accordingly, designers of playful processes may first and foremost focus on establishing an autonomy component, while the feeling of competence, also constitutional for intrinsic motivation, may be elicited in and by the process itself. Thus, if one designs for learning through play, one may want to pay particular attention to the engagement of participants and evaluate their experience of playfulness rather than focus on their *a priori* competences and on the game-like qualities of the situation.

### Limitations of Study and Directions for Future Research

We hope the results discussed above have demonstrated the usefulness of our approach for researchers interested in playfulness as a stance or state of mind. However, for a number of reasons, the study should be considered a pilot, and further empirical work may be required.

Firstly, our population sample comprises quite a homogenous group of students, all taking part of a schooling program (the Danish Hojskole) that in its creative curriculum might attract particularly playful participants. Unfortunately, we did not assess playfulness as a character trait specifically, e.g., using dedicated questionnaires (Proyer, 2012). Future research should explore if individuals, independent of their personal tendency to be playful, show behavior and experiential reports similar to the ones observed here.

Secondly, we may have primed some of the participants, by describing the non-playful condition as referring to a stance similar to “work.” The work-play contrast is indeed one that has been suggested by existing literature on play and might have triggered certain cultural connotations impinging on the otherwise data-driven approach (see for example Bateson and Martin, 2013). This term should thus be avoided in future replications of this work.

Thirdly, interviews revealed that the building material chosen for the experiment may in itself have primed participant to be playful. This could be for cultural as well as material reasons: LEGO bricks are deeply embedded in Danish culture and may obviously trigger childhood related memories in participants. As explicated above, it might also enhance explorative behavior due to its abstractness and sensual aesthetics. Future research should thus further explore the influence of the material chosen by comparing the results of this study with one using a material more neutral (that is not intuitively associated with either play or work) and possibly also with one clearly associated with work.

Fourthly, the data-driven analysis did not provide results that could immediately be ascribed to “relatedness,” the third component of intrinsic motivation according to Deci and Ryan’s theory. In this framework, “relatedness” is understood as a natural sense of belonging to the environment and the people around. One reason that this aspect of intrinsic motivation may not have shown up in the data, that otherwise strongly reminded of Deci and Ryan’s theory, is that the setup did not include obvious others – like building partners etc. It has been suggested that superiors like supervisors or teachers are important others too, with students being dependent on them to like, respect and value their work, and to develop intrinsic motivation for it. In this sense the experimenter might be interpreted as a distinct other. However, our data only gave indirect evidence of this, mainly with reference to an evaluative instance in the non-playful condition. Future research should engage in further exploring this aspect by modulating the setup to include partners in the task (see also Tylén et al., 2016).

### Summary

To the best of our knowledge, this is the first empirical and data-driven study assessing people’s experience of becoming playful. To our estimation our data shows that our experimental design and the chosen methods allow a deep and detail-rich insight into participants’ capacity of voluntary modulating their own playful stance.

In particular, interview results indicate that participants are able to voluntarily modulate their playfulness to the degree that they are able to modulate their autonomy in the building process and trust the process elicited by that: higher autonomy then facilitates a dynamic interaction with the material given, that can be further enhanced by the properties of that material. The surprising products of that activity make people aware of their own creative competence, which positively affects their mood and motivates them to continue the process. It seems that only experiences that fulfill these looping processes of autonomy, surprise, feeling of competence and motivation are categorized as playful in hindsight.

We thus propose a new working definition. Playfulness may be conceptualized as an attitude of throwing off constraints, which facilitates an explorative interaction with materials and others. This allows for intrinsic motivation to arise, supported by the surprising results of that interaction and by the connected positive emotions and feeling of competence.

When designing for playfulness, internal and external variables that modulate these processes should be taken into account.

We hope that future research will be able to make further usage of these findings and propositions.

## Author Contributions

KH designed the research, conducted and analyzed the data and wrote the manuscript. AR supervised all stages and reviewed the manuscript.

## Conflict of Interest Statement

The authors declare that the research was conducted in the absence of any commercial or financial relationships that could be construed as a potential conflict of interest.

## Footnotes

1 https://www.csullender.com/scholar/

2 http://www.danishfolkhighschools.com/about-folk-high-schools/history/
